# Effect of Foliar Application of Various Nitrogen Forms on Starch Accumulation and Grain Filling of Wheat (*Triticum aestivum L.*) Under Drought Stress

**DOI:** 10.3389/fpls.2021.645379

**Published:** 2021-03-25

**Authors:** Xiaokang Lv, Yunpeng Ding, Mei Long, Wenxin Liang, Xiaoyan Gu, Yang Liu, Xiaoxia Wen

**Affiliations:** College of Agronomy, Northwest A&F University, Yangling, China

**Keywords:** nitrogen, water deficit, grain weight, starch biosynthesis, hormone, antioxidant activity

## Abstract

Foliar nitrogen (N) fertilizer application at later stages of wheat (*Triticum aestivum L.*) growth is an effective method of attenuating drought stress and improving grain filling. The influences or modes of action of foliar application of various nitrogen forms on wheat growth and grain filling need further research. The objective of this study was to examine the regulatory effects of various forms of foliar nitrogen [NO_3_^–^, NH_4_^+^, and CO(NH_2_)_2_] on wheat grain filling under drought stress and to elucidate their underlying mechanisms. The relative effects of each nitrogen source differed in promoting grain filling. Foliar NH_4_^+^-N application notably prolonged the grain filling period. In contrast, foliar application of CO(NH_2_)_2_ and NO_3_^–^-N accelerated the grain filling rate and regulated levels of abscisic acid (ABA), z-riboside (ZR), and ethylene (ETH) in wheat grains. Analysis of gene expression revealed that CO(NH_2_)_2_ and NO_3_^–^-N upregulated the genes involved in the sucrose–starch conversion pathway, promoting the remobilization of carbohydrates and starch synthesis in the grains. Besides, activities of superoxide dismutase (SOD), peroxidase (POD), and catalase (CAT) were increased, whereas the content of malondialdehyde (MDA) declined under foliar nitrogen application (especially NH_4_^+^-N). Under drought stress, enhancement of carbohydrate remobilization and sink strength became key factors in grain filling, and the relative differences in the effects of three N forms became more evident. In conclusion, NH_4_^+^-N application improved the antioxidant enzyme system and delayed photoassimilate transportation. On the other hand, foliar applications of NO_3_^–^-N and CO(NH_2_)_2_ enhanced sink capacity and alleviated drought stress injury in wheat.

## Introduction

Drought is a severe source of abiotic stress that threatens wheat (*Triticum aestivum L.*) growth and yield worldwide and is exacerbated by climate change (Wang et al., 2016; Zhang et al., 2017; Alam et al., 2020). Northern China, a major global wheat-producing region, is frequently afflicted with drought during wheat growth, particularly when precipitation is relatively low and evapotranspiration is high in the spring (Deng et al., 2018; Jiang et al., 2020; Li et al., 2020). This leads to significant adverse effects on anthesis and grain filling (Li et al., 2000; Liu et al., 2014; Asif et al., 2016). As wheat production is vital to food security in China, there is an urgent need to mitigate the effects of drought stress on this crop.

As the core structural element of proteins, including enzymes and those involved in photosynthetic systems, nitrogen (N) is a key factor in crop growth and productivity (Beier et al., 2018; Evans and Clarke, 2018). N and water interact in significant ways that affect wheat growth. Water deficit impedes plant N uptake, causing a nitrogen deficiency which aggravates the damage caused by drought stress (Gonzalez-Dugo et al., 2010; Alam et al., 2020; Nazar et al., 2020). Efficacious N fertilizer management attenuates the effects of drought on wheat by maintaining normal crop physiology and scavenging reactive oxygen species (ROS) formed in response to drought stress (Agami et al., 2018; Guo et al., 2019). Wheat absorbs N in the form of ammonium (NH_3_^+^) and nitrate (NO_3_^–^) or organic N such as urea (Woolfolk et al., 2002; Carlisle et al., 2012). The latter is widely used as a nitrogen fertilizer in crop production in China (Tao et al., 2018). Previous studies have elucidated the relative effects of different forms of N fertilizer. Carlisle et al. (2012) reported that plants supplied with NH_4_^+^-N allocated comparatively more nutrients and biomass to the shoots, whereas those provided with NO_3_^–^-N distributed relatively more nutrients to the roots. NO_3_^–^-N stimulated root growth in rice with high nitrogen use efficiency (NUE). In this way, biomass accumulation and NUE were enhanced at later growth stages (Song et al., 2011). Other studies reported that various forms of N have different effects on plant stress resistance. In rice, NH_4_^+^-N supplementation mitigates cadmium stress by inhibiting the uptake and transport of the metal. NH_4_^+^-N also increased rice and maize drought tolerance (Smiciklas and Below, 1992; Gao et al., 2009; Wu et al., 2018; Zhu et al., 2018). In contrast to the numerous studies focusing on the effects of various forms of exogenous N on root growth and nutrient element transport, research on the effects of various forms of N applied under abiotic stress on wheat grain filling is relatively sparse.

Grain filling is an intensive endogenous nutrient transport process regulated by soil moisture content and phytohormones (Chu et al., 2015). When drought stress occurs between the period of anthesis and grain filling, wheat yield decreases sharply (Zhang et al., 2017). Grain filling mainly involves starch biosynthesis, as starch comprises 60–80% of the grain weight (Wang Z. et al., 2014). Starch biosynthesis comprises a series of enzyme-catalyzed reactions in the grain (Emes et al., 2003). According to Tetlow (2006), soluble starch synthases (SSS, EC 2.4.1.21) catalyze chain elongation; granule-bound starch synthases (GBSS, EC 2.4.1.21) form amylose; and soluble starch synthases elongate amylopectin glucan chains. The branch points in starch molecules are introduced by starch branching enzymes (SBE, EC 2.4.1.18). Amylopectin is synthesized by the coordinated actions of SSS and SBE (Tetlow, 2006). Based on our previous research and other reports, wheat grain filling may be regulated by multiple hormones (Xu et al., 2007; Lv X. et al., 2017; Lv X. K. et al., 2017). Higher ABA and lower ethylene concentrations are associated with higher grain filling rates (Yang et al., 2006; Chu et al., 2015). Drought stress reduces the levels of Z-riboside (ZR) and abscisic acid (ABA) and increases ethylene (ETH) evolution (Lv X. K. et al., 2017; Yaseen et al., 2020). This stressor may also trigger membrane lipid peroxidation; induce malondialdehyde (MDA) accumulation; downregulate catalase (CAT), peroxidase (POD), and superoxide dismutase (SOD); and accelerate plant senescence (Yamazaki and Kamimura, 2002; Liu et al., 2016; Lv X. K. et al., 2017). The effects of various forms of N on wheat grain filling and their mechanisms under drought stress are still unclear.

Many investigations explored the effect of nutrients on wheat grain filling through foliar application, as leaves were more efficient at absorbing nutrients during the later stages of wheat growth compared with senescent roots (Kutman et al., 2010; Uscola et al., 2014; Visioli et al., 2018). Nevertheless, researches on the effect of foliar application of various forms of nitrogen on wheat grain filling were sparse. Thus, the present study aimed to investigate the regulatory effects of foliar NO_3_^–^, NH_4_^+^, and CO(NH_2_)_2_ on wheat grain filling and starch accumulation under drought stress. For this purpose, foliar N application was conducted at anthesis, and drought stress was induced from anthesis to maturity. To elucidate the underlying mechanism, we evaluated how different N sources regulated endogenous phytohormones, the antioxidant enzyme system in flag leaves, and gene expression involved in grain starch biosynthesis. Results in this work could provide insight relevant to (i) the regulatory effects of different N sources on grain filling and starch accumulation; (ii) the response of starch biosynthesis, phytohormones levels, and plant senescence to different N sources; and (iii) the alleviative effect of different N sources on drought stress of wheat during grain filling period. This research might be valuable for wheat production under climate change.

## Materials and Methods

### Study Site and Treatment Descriptions

A pot experiment using a split-plot test design was conducted in large waterproof sheds at the experimental station of the Agricultural Crop Specimen Area of Northwest A&F University, Shaanxi Province, China (elevation: 466.7 m; mean annual temperature: 12.9°C). Before putting into pots, the soil was crushed and sifted away plant residue, thereby preventing soil hardening and facilitating nutrient absorption. Each pot had a diameter and height of 24.25 and 26.4 cm, respectively, and was filled with 25 kg soil. The readily available levels of N, P, and K were 51.23, 20.01, and 105.37 mg kg^–1^, respectively. A total of 240 pots were used in the experiment. Before planting, 3.89 g urea and 1.29 g monopotassium phosphate were applied to the soil in each pot. The wheat (*Triticum aestivum L.*) cultivar Xinong979, a cultivar currently grown in Huanghuai wheat production region of China, was planted in each pot on October 9, 2015. The seeds were pre-washed with 3% (v/v) H_2_O_2_ and soaked in water for 24 h. A total of eight treatment regimens were performed, each comprising 30 pots with 15 seeds placed near the center of each. The soil was irrigated until flowering in order to maintain normal water potential at the 15-cm soil layer (−20 ± 5 kPa).

From anthesis to maturity, two soil moisture levels were set up by controlling the irrigation rates and volumes. For the well-watered (WW) and soil-dried (SD) treatments, the water potentials at the 15-cm soil layer were maintained at -20 ± 5 and -60 ± 5 kPa, respectively. Water potential was monitored and recorded between 11:00 and 12:00 daily using tensiometers (SWP-100; Soil Science Research Institute, China Academy of Sciences, Nanjing, China) installed in each pot. Under each treatment at anthesis, aqueous solutions of urea [CO(NH_2_)_2_], NaNO_3_ (NO_3_^–^), or NH_4_Cl (NH_4_^+^) were sprayed on the leaves at the rate of 750 kg ha^–1^ for 3 d, which meant 3.46 g pot^–1^ according the pot’s surface area. The concentrations of urea, NaNO_3_, and NH_4_Cl were 3.0, 8.5, and 5.3%, respectively. These concentrations ensured that the total N content was equal for all three types of N fertilizer; 0.01%(V/V) Tween-20 was added into each solution. Equal volumes of deionized water were applied to the control plants (CK1, CK2) under well-watered (WW) and soil-dried (SD) treatments separately.

### Sampling and Measurement

Wheat spikes flowered at the same day were labeled for sampling. Twenty spikes were sampled at 4-d intervals from anthesis to maturity. All of the grains on each spike were removed. Half of the grain samples were frozen in liquid N. Subsequently, samples used to measure the phytohormone levels were stored at −40°C, and samples used to quantity the expression levels of the genes were stored at −80°C. The other half of the grain samples was dried at 70°C and used to determine the constant weight and the sucrose and starch content. On a single day, 20 flag leaves were sampled from each pot, frozen in liquid nitrogen, stored at −40°C, and used to measure superoxide dismutase (SOD, EC 1.15.1.1), peroxidase (POD, EC 1.11.1.7), and catalase (CAT, EC 1.11.1.6) activity and malondialdehyde (MDA) content.

### Grain-Filling Process

The grain filling process was fitted to Richards’s (1959) growth equation according to Yang et al. (2006):

(1) $$W=\frac{A}{{\left(1+B\,e^{-k\,t}\right)}^{\frac{1}{N}}}$$

The grain filling rate (G) was calculated as follows:

(2) $$G=\frac{A\,k\,B\,{\text{e}}^{-k\,t}}{{\left(1+B\,{\text{e}}^{-k\,t}\right)}^{\left(\frac{N+1}{N}\right)}}$$

where W is the grain weight (mg); A is the final weight (mg); t is the time after anthesis (d); and B, k, and N are coefficients determined by regression.

The active grain filling period is that in which W is between 5% (*t*_1_) and 95% (*t*_2_) of A. The average grain filling rate during this period was calculated for the interval between *t*_1_ and *t*_2_.

### Sucrose, Amylose, and Amylopectin Content

Dried grain samples were pulverized for analysis of sucrose, amylose, and amylopectin levels. Then, 0.2 g of the powder was extracted with 6 mL of 80% (v/v) ethanol for 30 min in a water bath at 80°C. The suspension was centrifuged at 5,000 × g for 10 min at 25°C, and the supernatant was collected. The extraction was performed in triplicate. The three supernatants were pooled and diluted with 80% (v/v) ethanol to achieve a volume of 25 mL for sucrose measurement. The sucrose content was determined by the resorcinol method. Absorbance was read at 480 nm, and the sucrose content was interpolated from a standard curve (Wang B. S. et al., 2015). Sample powder (0.1 g) was stirred in a water bath with 10 mg of 0.5 M KOH for 30 min at 90°C. After the solution was diluted to a volume of 50 mL with distilled water, 2.5 mL was transferred to a fresh tube containing 20 mL distilled water. The pH was adjusted to 3.5 with 0.1 M HCl; 0.5 mL I_2_-KI reagent was added, and the solution was diluted to 50 mL with distilled water. After 20 min, the absorbance of the solution was measured at wavelengths of 631, 480, 554, and 754 nm. The amylose and amylopectin levels in the wheat grains were determined according to the method described by Jiang et al. (2003). The total starch content is the sum of amylose content and amylopectin content.

### Phytohormones

Extraction and purification of Z+ZR and ABA were carried out according to the methods described by Yang J. et al. (2001). Samples ∼0.5 g in mass were ground in a mortar on ice and homogenized with 5 mL of 80% (v/v) methanol containing 1 mM butylated hydroxytoluene (BHT) as an antioxidant. The extracts were incubated for 4 h at 4°C and centrifuged at 10,000 × g and 4°C for 15 min. The supernatants were passed through a Chromosep C18 column (C18 Sep-Park Cartridge; Waters Corp, Milford, MA, United States) in order to isolate the antioxidant enzymes. The fractions were vacuum-dried at 40°C and dissolved in 1 mL phosphate-buffered saline (PBS) containing 0.1% (w/v) gelatin (pH 7.5) and 0.1% (v/v) Tween 20 for an enzyme-linked immunosorbent assay (ELISA; Phytohormones Research Institute, China Agricultural University, Beijing, China). Levels of Z+ZR and ABA were measured by ELISA using previously described methods (Yang J. et al., 2001).

Ethylene (ETH) evolution from wheat grains was measured according to Yang et al. (2006). The ETH was assayed by gas chromatography (GC) (Trace GC UItra^TM^; Thermo Fisher Scientific, Waltham, MA, United States) according to a previous study (Lv X. K. et al., 2017).

### SOD, POD, CAT, and MDA Content in Flag Leaves

One half gram of fresh flag leaves was ground in a mortar filled with 5 mL extraction buffer comprising 100 mM potassium phosphate buffer (pH 7.0), 1% (w/v) polyvinylpyrrolidone (PVPP), and 1 mM ethylenediaminetetraacetic acid (EDTA). After centrifugation at 20,000 rpm and 4°C for 20 min, the supernatant was collected for antioxidant enzyme analysis. The activity of superoxide dismutase (SOD; EC 1.15.1.1) was assayed by the inhibition of nitro blue tetrazolium (NBT) photoreduction as previously described (Wang et al., 2021). The optical density of the product was measured at 560 nm. One unit of SOD corresponded to the amount of enzyme inhibiting 50% of the NBT photoreduction. Catalase (CAT; EC 1.11.1.6) activity was determined from H_2_O_2_ decomposition over a 3-min interval. Absorbance of the product was measured at 240 nm as previously reported (Wang et al., 2021). Guaiacol peroxidase (POD; EC 1.11.1.7) activity was evaluated by guaiacol oxidation. Absorbance of the product was read at 470 nm according to the method described by Wang and Huang (2000).

Based on a previous study (Lv X. K. et al., 2017), 0.5 g of leaf tissue was homogenized in 5 mL of 0.1% (w/v) trichloroacetic acid (TCA) and centrifuged at 20,000 × g and 4°C for 20 min. The MDA content was determined by the method of Wang and Huang (2000) using the following equation:

(3) MDA⁢(η⁢M)=[(A535-A600)/1.56]×105

### RT-PCR of the Genes Encoding the Enzymes Involved in Starch Synthesis

A reverse-transcriptase polymerase chain reaction (RT-PCR) was performed to evaluate the relative expression levels of the genes encoding the enzymes participating in starch biosynthesis, including adenosine diphosphate pyrophosphorylase (AGPP), granule-bound starch synthase (GBSS), soluble starch synthase (SSS), and starch branching enzyme (SBE). Total RNA from wheat grain was isolated with an E.Z.N.A. plant RNA kit (Omega Bio-Tek Inc., Norcross, GA, United States) according to the manufacturer’s instructions. RNA concentration and quality were measured with a NanoDrop^TM^ 2000 spectrophotometer (NanoDrop Technologies, Wilmington, DE, United States). RNA was reverse transcribed with a PrimeScriptTMRT reagent kit (TaKaRa Bio Inc., Shiga, Japan). The cDNA product was subjected to real-time PCR (QuantStudio 3; Applied Biosystems, Foster City, CA, United States) using a two-step method and a SYBR premix Ex Taq II kit (TaKaRa Bio Inc., Shiga, Japan). The conserved regions of the gene sequences of *AGPP-L, GBSS-I, SSS-1, SSS-II, SSS-III, SBE-I, SBE-IIa*, and *SBE-IIb* were obtained from wheat and used to design primers for the detection of gene expression in wheat grain. The gene-specific primers and the base pair (bp) sizes of the fragments generated are listed in Supplementary Table 1. The transcript levels of the selected genes were measured by qPCR on a QuantStudio 3 real-time PCR system (Applied Biosystems, Foster City, CA, United States) with a SYBR premix Ex Taq II kit (TaKaRa Bio Inc., Shiga, Japan). Each reaction consisted of 25 μL SYBR premix Ex Taq^TM^ (2X), 4 μL diluted cDNA, 2 μL forward primer, 2 μL reverse primer, 1 μL Rox Reference Dye II, and 16 μL ddH_2_O in a total volume of 50 μL. The relative transcription levels of the starch synthesis-related enzyme genes were calculated using the 2^–ΔΔ*Ct*^ method (Han et al., 2006). Wheat β-actin (GenBank Accession No. AB181991) were used as internal controls.

The data were analyzed for variance in SPSS v. 16.0 for Windows (IBM Corp., Armonk, NY, United States). Means were tested by the least significant difference method. *P* < 0.05 was considered statistically significant.

## Results

### Grain Filling

**FIGURE 1:**
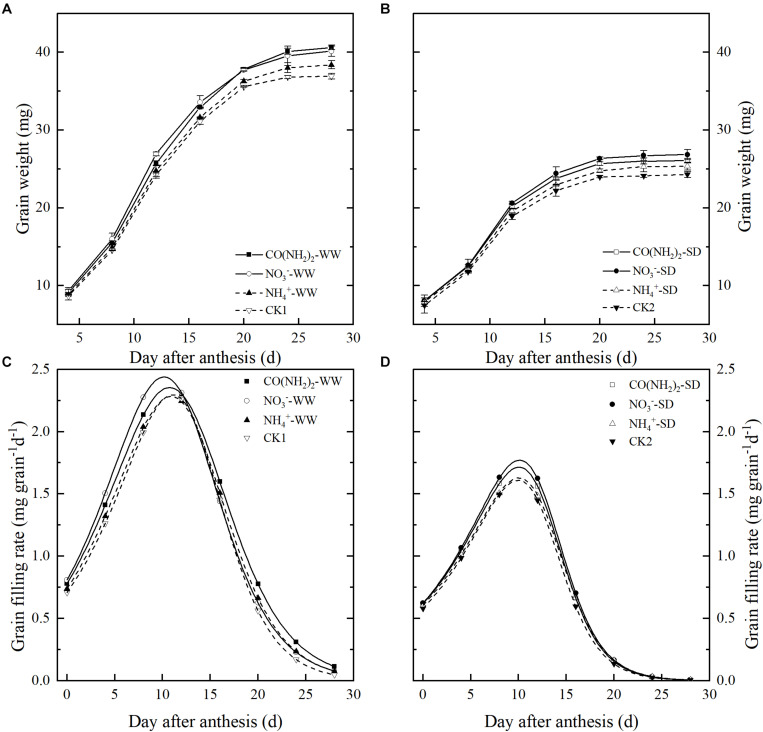
Effect of N forms in foliar fertilizer on grain weights [**(A)** well-watered treatment, WW; **(B)** soil-dried treatment, SD] and grain filling rates [**(C)** well-watered treatment, WW; **(D)** soil-dried treatment, SD]. Vertical bars represent ± the standard deviation of the mean (*n* = 3). CO(NH_2_)_2_, NO_3_^–^, and NH_4_^+^ represent CO(NH_2_)_2_, NaNO_3_, and NH_4_Cl, whose concentration was described in the Materials and Methods section. CK1 and CK2 mean that deionized water was sprayed on leaves at anthesis under well-watered treatment and soil-dried treatment, respectively.

Under the WW treatment, foliar application of all N sources enhanced grain filling and increased the final grain weight. However, the various foliar N applications had different effects on the grain filling characteristics (Figure 1 and Table 1). Compared with CK1, the CO(NH_2_)_2_ application significantly increased the maximum and mean grain filling rates and extended the grain filling period. Application of NO_3_^–^-N did not influence duration of the grain filling period, but resulted in the highest maximum and mean grain filling rates. The final grain weight of NO_3_^–^-N was similar to the CO(NH_2_)_2_ application. Application of NH_4_^+^-N did not affect the maximum or mean grain filling rates, but prolonged the active grain filling period compared with CK1 and NO_3_^–^-N. The final grain weight under the NH_4_^+^-N application was greater than that of CK1. The effect of NH_4_^+^-N on the maximum and mean grain filling rate was similar to that obtained with CO(NH_2_)_2._

**TABLE 1:** Effect of N forms in foliar fertilizer on grain filling characteristics of wheat.

**Soil moisture**	**Treatment**	**Wmax mg**	**Gmax mg grain^–1^d^–1^**	**Gmean mg grain^–1^d^–1^**	**D d**
WW	CO(NH_2_)_2_	41.15 a	2.35b	1.55 b	26.55 a
	NO_3_^–^	40.47 a	2.44a	1.61 a	25.14 b
	NH_4_^+^	38.72 b	2.29bc	1.50 c	25.87 a
	CK1	37.39 c	2.27c	1.47 c	25.38 b
SD	CO(NH_2_)_2_	26.01 b	1.71b	1.10 b	23.92 b
	NO_3_^–^	26.70 a	1.78a	1.15 a	23.62 b
	NH_4_^+^	25.46 c	1.62c	1.01 d	24.81 a
	CK2	24.47 d	1.64c	1.04 c	23.48 b

*Values with a column and for the same soil moisture followed by the same letters are not significantly different at *P < 0.05.* WW means well-watered treatment and SD means soil-dried treatment. CK1 and CK2 mean that deionized water was sprayed on leaves at anthesis under well-watered treatment and soil-dried treatment, respectively. CO(NH_2_)_2_, NO_3_^–^, and NH_4_^+^ represent CO(NH_2_)_2_, NaNO_3_, and NH_4_Cl, whose concentration was described in the Materials and Methods section. Wmax, Gmax, Gmean, and D mean that the final grain weight, the maximum grain-filling rates, the mean grain-filling rates, and active grain-filling period, respectively.*

The soil-drying treatment markedly inhibited grain filling and resulted in a loss of grain weight (Figure 1 and Table 1). The NO_3_^–^-N treatment achieved the highest maximum and mean grain filling rates, whose final grain weight was the highest. The CO(NH_2_)_2_ treatment increased the maximum and mean grain filling rates and extended the active grain filling period, though not to a significant extent. The final grain weight under CO(NH_2_)_2_ was lower than that for the NO_3_^–^-N treatment. There was only the NH_4_^+^-N treatment extended the active grain filling period compared with other treatments and control. The increase of grain weight on the NH_4_^+^-N treatment was lower than the CO(NH_2_)_2_ and NO_3_^–^-N treatments. Whereas the NH_4_^+^-N treatment decrease the mean grain filling rate and had no significant effect on the maximum grain filling rate compared with the CK under drought stress.

### Sucrose, Amylose, Amylopectin, and Total Starch Content

**FIGURE 2:**
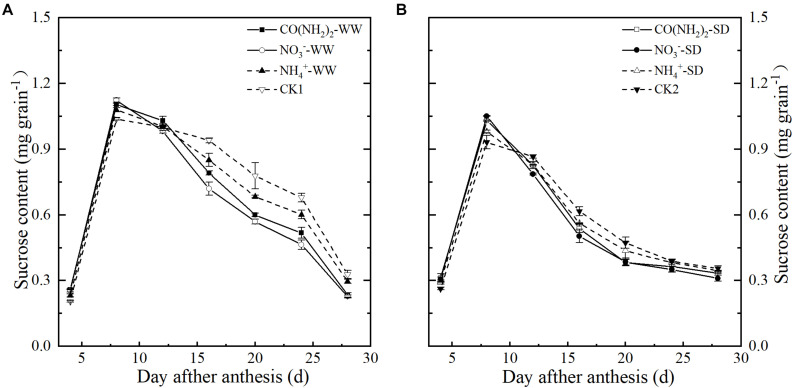
Effect of N forms in foliar fertilizer on the sucrose content in the wheat grains [**(A)** well-watered treatment, WW; **(B)** soil-dried treatment, SD]. Vertical bars represent ± the standard deviation of the mean (*n* = 3). CO(NH_2_)_2_, NO_3_^–^, and NH_4_^+^ represent CO(NH_2_)_2_, NaNO_3_, and NH_4_Cl, whose concentration was described in Materials and Methods. CK1 and CK2 mean that deionized water was sprayed on leaves at anthesis under well-watered treatment and soil-dried treatment, respectively.

Under the WW treatment, sucrose levels increased in the early grain filling stages and peaked at 8 d post-anthesis (Figure 2). Compared with CK1, the foliar CO(NH_2_)_2_ and NO_3_^–^-N applications enhanced sucrose accumulation during the early grain filling stage and reduced the sucrose content at the late grain filling stage. At 8 d post anthesis, sucrose content in grains under the NO_3_^–^-N applications was the highest. The sucrose content in NO_3_^–^-N applications was lower than that in CO(NH_2_)_2_ application from 12 d to 24 d post anthesis. Sucrose content under the NH_4_^+^-N treatment was higher that under the CO(NH_2_)_2_ and NO_3_^–^-N treatments from 16 d post anthesis. The trends in grain sucrose content were similar for WW and SD, whereas the sucrose content in grains approached the final level earlier under SD. The effects of different N forms on the changes of sucrose content under SD were similar to those under WW, while the difference between the foliar CO(NH_2_)_2_ and NO_3_^–^-N applications was smaller. There was no significance difference between the foliar CO(NH_2_)_2_ and NO_3_^–^-N applications from 16 d to 28 d post anthesis. Compared with CK2, the NH_4_^+^-N treatment did not change the sucrose levels significantly from 20 d to 28 d post anthesis.

**FIGURE 3:**
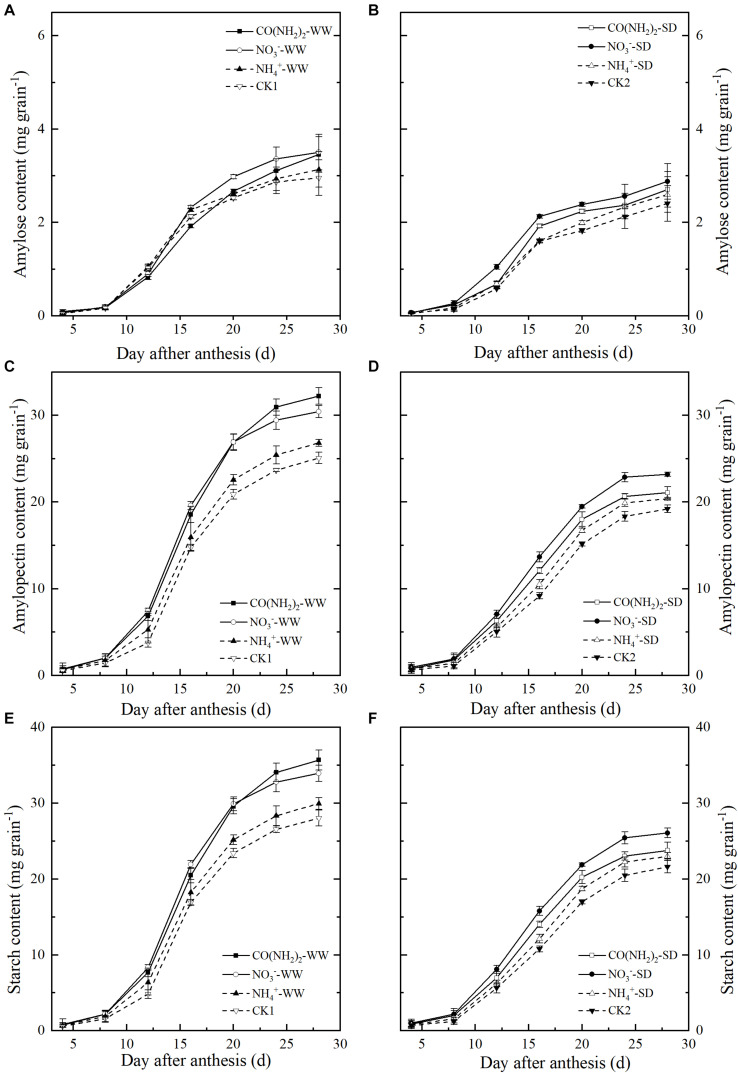
Effect of N forms in foliar fertilizer on the amylose content, amylopectin content, and total starch content in the wheat grains [**(A,C,E)** well-watered treatment, WW; **(B,D,F)** soil-dried treatment, SD]. Vertical bars represent ± the standard deviation of the mean (*n* = 3). CO(NH_2_)_2_, NO_3_^–^, and NH_4_^+^ represent CO(NH_2_)_2_, NaNO_3_, and NH_4_Cl, whose concentration was described in the Materials and Methods section. CK1 and CK2 mean that deionized water was sprayed on leaves at anthesis under well-watered treatment and soil-dried treatment, respectively.

Total starch, amylose, and amylopectin accumulation increased during grain filling (Figure 3). Starch and amylopectin levels were significantly lower under the SD treatment than the WW treatment; no significant differences were found in amylose levels (Figures 3A,B). Amylopectin was the main component of starch in grains. In this experiment amylopectin and total starch had similar trends. The effects of foliar N application on total starch and amylopectin content resembled those observed for grain filling (Figures 3B–F). Under WW, the total starch and amylopectin levels in grains sprayed with CO(NH_2_)_2_ were lower than those in plants sprayed with NO_3_^–^-N at the early and middle grain filling stages. At the late grain filling stage, however, total starch and amylopectin were higher in CO(NH_2_)_2_-sprayed plants than they were in NO_3_^–^-N-treated plants. Under SD, the total starch and amylopectin levels were highest in the plants sprayed with NO_3_^–^-N.

### Changes in Phytohormone Level

**FIGURE 4:**
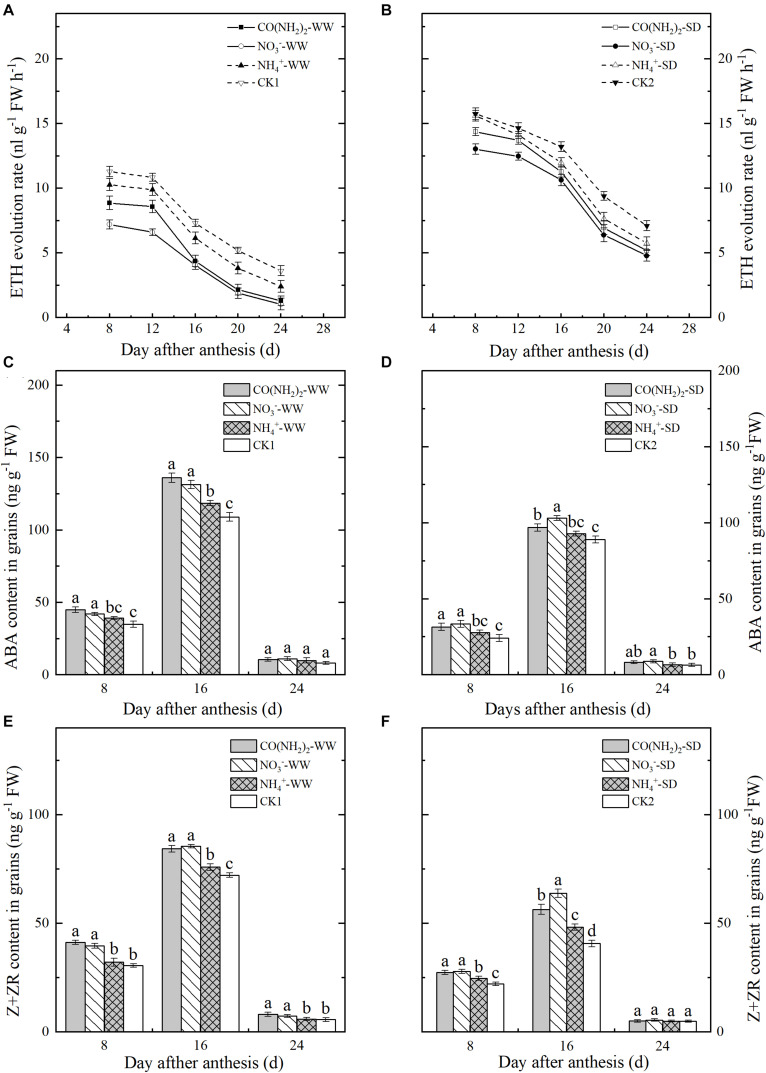
Effect of N forms in foliar fertilizer on the ETH revolution rate, ABA, and Z+ZR content in the wheat grains [**(A,C,E)** well-watered treatment, WW; **(B,D,F)** soil-dried treatment, SD]. Vertical bars represent ± the standard deviation of the mean (*n* = 3). Values followed by the same letters in each soil moisture are not significantly different at *P* < 0.05 level. CO(NH_2_)_2_, NO_3_^–^, and NH_4_^+^ represent CO(NH_2_)_2_, NaNO_3_, and NH_4_Cl, whose concentration was described in the Materials and Methods section. CK1 and CK2 mean that deionized water was sprayed on leaves at anthesis under well-watered treatment and soil-dried treatment, respectively.

The rate of ETH evolution steadily declined in grains during the filling stage (Figures 4A,B). The SD treatment significantly enhanced ETH evolution even at the late grain filling stage. The relative effects of the various foliar N applications on ETH evolution were similar under both water regimes. However, the ETH evolution rates were significantly lower for the NO_3_^–^-N and CO(NH_2_)_2_ treatments than the others. At the early and middle grain filling stages, the NH_4_^+^-N treatment had no significant influence on ETH evolution. At the late grain filling stage, addition of NH_4_^+^-N decreased ETH evolution compared to CK.

Changes in grain ABA and Z+ZR content followed similar trends and peaked by the middle grain filling stage. Drought stress decreased ABA and Z+ZR in grains. Under CK2, ABA and Z+ZR were lowered by 18.38 and 43.61%, respectively, compared to CK1 at the middle grain filling stage (Figures 4C–F). Foliar NH_4_^+^-N fertilizer did not significantly influence the ABA and Z+ZR levels at the early and late grain filling stages except for Z+ZR under the SD treatment. The NO_3_^–^-N and CO(NH_2_)_2_ treatments significantly increased ABA and Z+ZR except at the late grain filling stage. Levels of ABA and Z+ZR were relatively higher under the SD treatment at the middle grain filling stage.

### Relative Expression of Genes Encoding Enzymes Involved in Starch Synthesis

**FIGURE 5:**
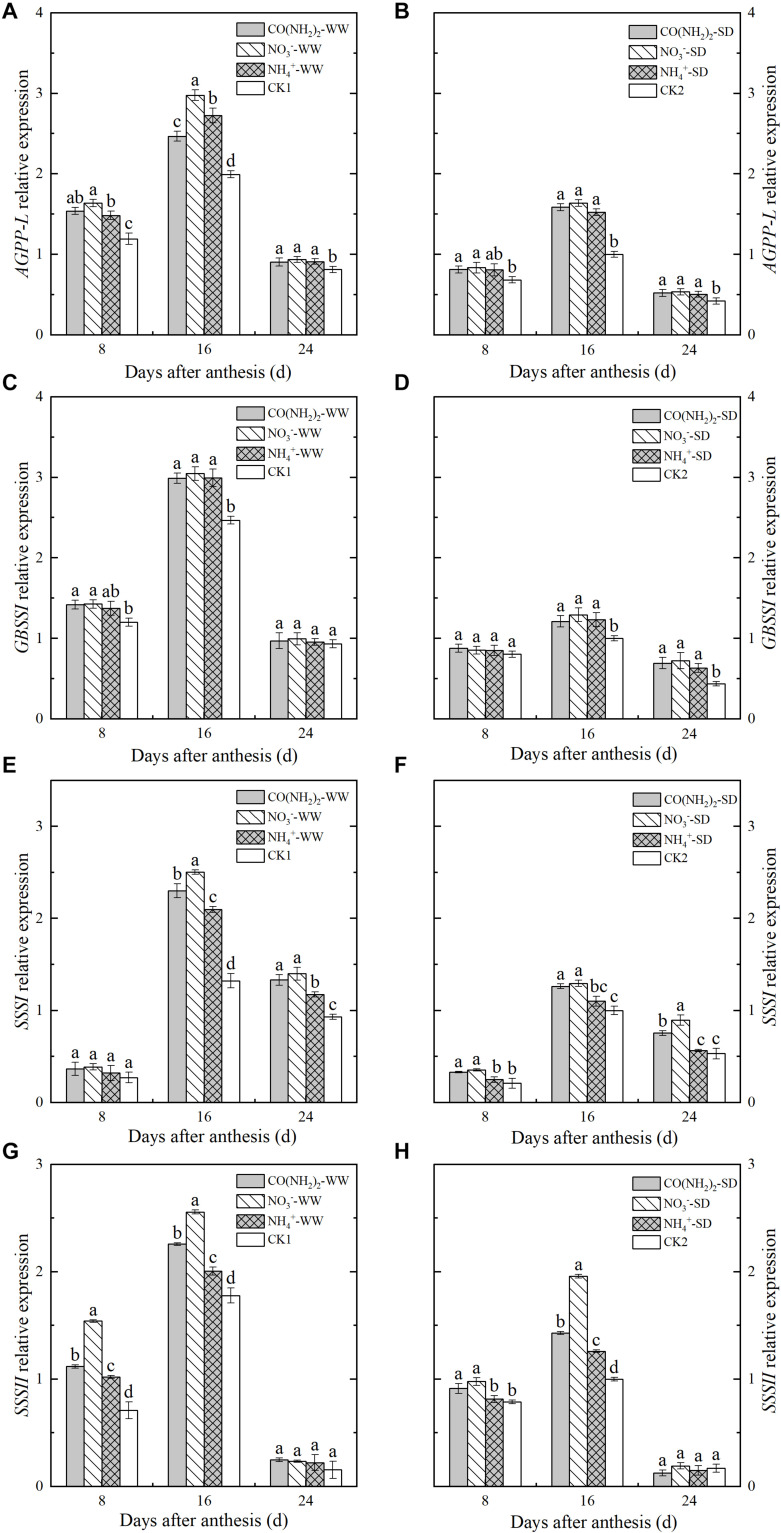
Effect of N forms in foliar fertilizer on the relative expression of *AGPPL, GBSSI, SSSI*, and *SSSII* enzyme genes in the wheat grains [**(A,C,E,G)** well-watered treatment, WW; **(B,D,F,H)** soil-dried treatment, SD]. Vertical bars represent ± the standard deviation of the mean (*n* = 3). CO(NH_2_)_2_, NO_3_^–^, and NH_4_^+^ represent CO(NH_2_)_2_, NaNO_3_, and NH_4_Cl, whose concentration was described in the Materials and Methods section. CK1 and CK2 mean that deionized water was sprayed on leaves at anthesis under well-watered treatment and soil-dried treatment, respectively. Values followed by the same letters in each soil moisture are not significantly different at *P < 0.05* level.

**FIGURE 6:**
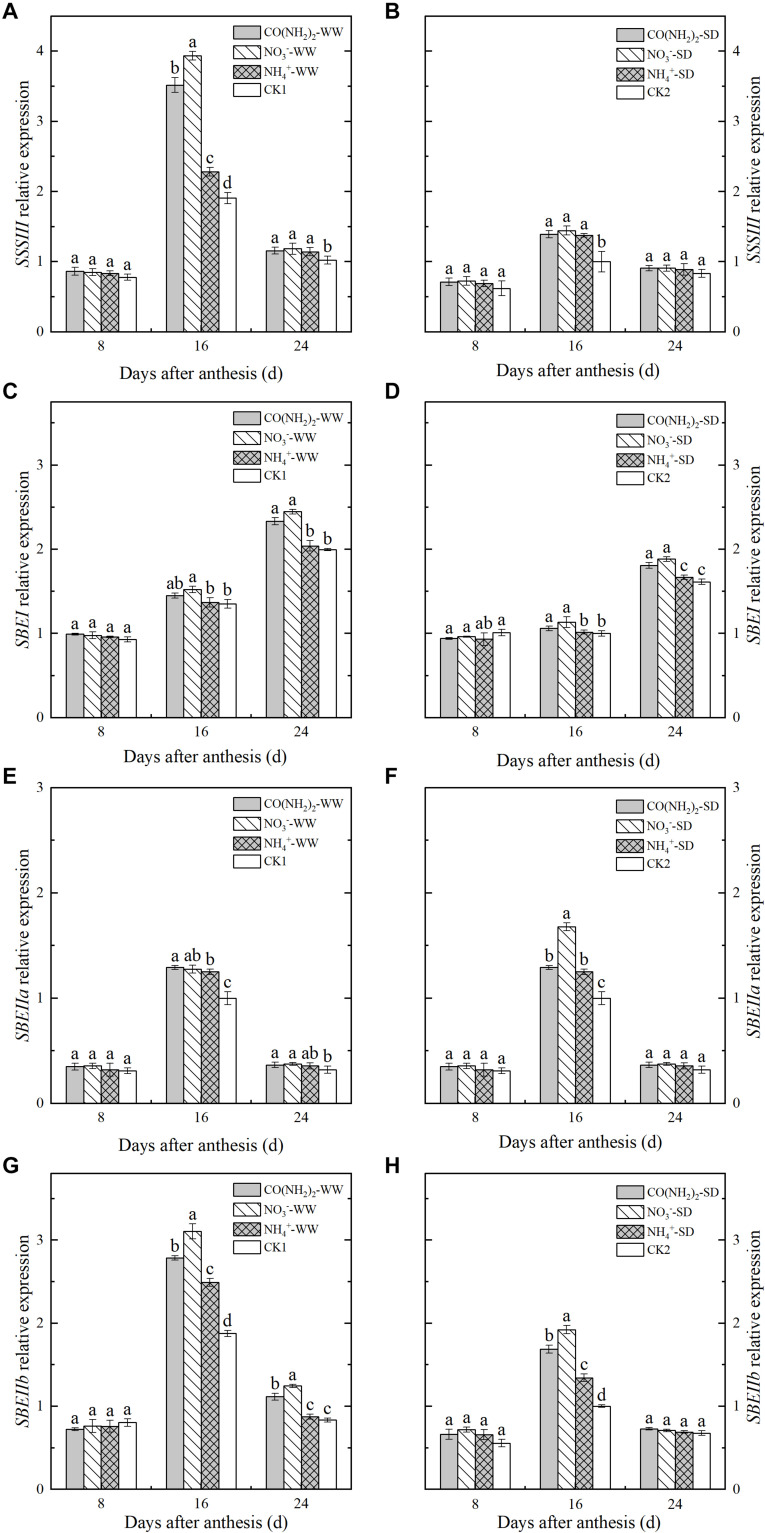
Effect of N forms in foliar fertilizer on the relative expression of *SSSII, SBEI, SBEIIa*, and *SBEIIb* enzyme genes in the wheat grains [**(A,C,E,G)** well-watered treatment, WW; **(B,D,F,H)** soil-dried treatment, SD]. Vertical bars represent ± the standard deviation of the mean (*n* = 3). CO(NH_2_)_2_, NO_3_^–^, and NH_4_^+^ represent CO(NH_2_)_2_, NaNO_3_, and NH_4_Cl, whose concentration was described in the Materials and Methods section. CK1 and CK2 mean that deionized water was sprayed on leaves at anthesis under well-watered treatment and soil-dried treatment, respectively. Values followed by the same letters in each soil moisture are not significantly different at *P < 0.05* level.

Analysis of the relative expression levels of the genes encoding enzymes involved in starch synthesis revealed that drought stress downregulated nearly of all these and blocked starch biosynthesis (Figures 5, 6). Foliar application of the various N fertilizers upregulated the *AGPP-L* and *GBSSI* genes. However, their expression levels did not significantly differ from each other except for *AGPP-L* under the WW treatment during the middle grain filling stage (Figures 5A,B). The *SSSI* and *SSSII* genes, both of which encode SSS, were expressed at low levels during the early and late grain filling stages (Figures 5C–F). At the middle grain filling stage, the foliar NO_3_^–^-N and CO(NH_2_)_2_ treatments upregulated *SSSI* and *SSSII.* Treatment with NO_3_^–^-N was comparatively more effective at lowering the expression of these genes than the other N forms. Expression of *SSSIII* was maximum during the middle grain filling stage. The effects of the various forms of N on relative *SSSIII* expression were similar to those for *SSSI* and *SSSII* (Figures 6A,B).

Changes in expression level differed among the three genes encoding SBE. The relative expression of *SBEI* continued to increase over the whole grain filling period, whereas *SBEIIa* and *SBIIIb* were expressed at their maximum levels during the middle grain filling stage and declined thereafter. The NH_4_^+^-N treatment did not significantly affect the relative *SBEI* expression, but upregulated *SBEIIa* and *SBEIIb* during the middle grain filling stage (Figures 6C,D). The expression levels of *SBEIIa* and *SBEIIb* under the NO_3_^–^-N and CO(NH_2_)_2_ treatments were higher than those under CK. Maximum expression of these genes occurred during the middle grain filling period.

### Antioxidant Enzymes and MDA

**FIGURE 7:**
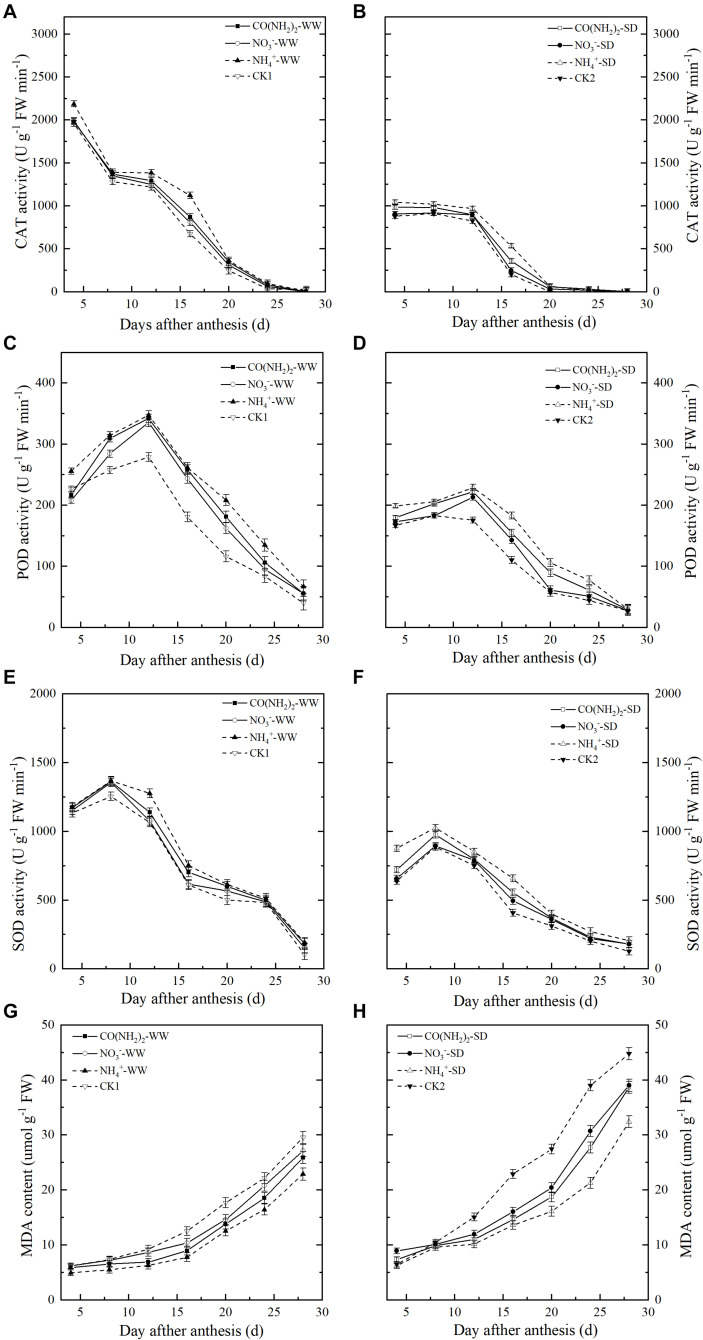
Effect of N forms in foliar fertilizer on the CAT, POD, and SOD activity and MDA content in the wheat flag leaves [**(A,C,E,G)** well-watered treatment, WW; **(B,D,F,H)** soil-dried treatment, SD]. Vertical bars represent ± the standard deviation of the mean (*n* = 3). CO(NH_2_)_2_, NO_3_^–^, and NH_4_^+^ represent CO(NH_2_)_2_, NaNO_3_, and NH_4_Cl, whose concentration was described in Materials and Methods. CK1 and CK2 mean that deionized water was sprayed on leaves at anthesis under well-watered treatment and soil-dried treatment, respectively.

During the grain filling stage, the CAT activity in the flag leaves steadily decreased (Figures 7A,B). In contrast, POD and SOD activity levels in the flag leaves increased until the middle grain filling stage, peaked at 12 d and 8 d post-anthesis, respectively, then decreased thereafter (Figures 7C–F). Drought stress downregulated CAT, POD, and SOD. Three forms of N application had positive effects on activities of antioxidant enzymes, while the NO_3_^–^-N treatment had relatively less influence on these enzymes during most of the grain filling process. Effects of CO(NH_2_)_2_ treatments on these antioxidants were slightly stronger than that under the NO_3_^–^-N treatment. Activities of CAT, POD, SOD under NH_4_^+^-N treatments were the highest during most of the grain filling period.

The MDA content of the flag leaves continuously increased during the grain filling period (Figures 7G,H). Drought stress promoted MDA accumulation whereas foliar N application decreased the MDA content. Difference between three forms treatment was significant, which was amplified under SD. The MDA levels in the plants treated with foliar NH_4_^+^-N was the lowest than those under other treatments throughout grain filling. The MDA levels under CO(NH_2_)_2_ treatment was substantially lower than that under the NO_3_^–^-N treatment during most of the grain filling period.

## Discussion

As an economical and efficient method widely practiced in China, foliar application of fertilizer can supply nutrient for crops more quickly than soil application, especially at the late growth period of crops when root activity keeps decreasing (Fageria et al., 2009; Zhang et al., 2010). Previous studies showed that foliar application of urea is beneficial for wheat growth (Blandino et al., 2015; Wang et al., 2021). We thus used this method of fertilization to determine specific effects of various forms of N on grain filling under well-watered and drought stress condition. In addition to damaging roots and above-ground biomass (Djanaguiraman et al., 2018), severe soil drought also impedes the grain filling process, thereby adversely affecting grain yield (Barnabas et al., 2008). In alignment with most references (Farooq et al., 2014; Liu et al., 2016) our results showed that soil drought stress decreased the final grain weight sharply. One study that produced contradictory results was that of Ramadan (Agami et al., 2018), in which the grain filling capacity increased under water deficit treatment. One possible reason is that only moderate drought stress, which is known contribute to increased grain weight if properly controlled, was induced in that study (Yang and Zhang, 2006).

Grain filling rate and duration both contribute to the final grain weight in wheat (Lv X. et al., 2017). All types of N fertilizer tested in this study significantly increased grain weight relative to CK. Based on our observation that these treatments had different effects on grain filling characteristics, we posit that grain weight was increased via different mechanisms for each. Although there is limited information available on the effects of various forms of N fertilizer on grain filling, our results indicate that foliar NO_3_^–^-N applications may improve grain filling and alleviate drought stress damage by accelerating the maximum and mean grain filling rates, whereas foliar NH_4_^+^-N application may extend the grain filling period. Foliar CO(NH_2_)_2_ application may have yielded the largest final grain weight under well-watered conditions by affecting the grain filling rate and period in a coordinated way. However, the coordination effect appears to be weakened by drought, leading to a lower grain weight for the foliar CO(NH_2_)_2_ application than for NO_3_^–^-N. This observation is supported by studies that have reported that the benefits of accelerated grain filling rate outweighed those of extended grain filling periods on final grain weight (Yang and Zhang, 2006; Yang et al., 2006). This explanation is consistent with the lower grain weight produced under the NH_4_^+^-N than the other two forms of N.

Comprising > 65% of the grain weight, starch is the main determinant of both grain weight and yield (Kumar et al., 2018). Biosynthesis of starch from sucrose is the main contributor to grain filling (Yang et al., 2004a; Wang Z. et al., 2014). Our results concur with previous studies, which report that the starch content and grain weight vary proportionally during grain filling (Dai et al., 2009; Zi et al., 2018). Earlier studies have also reported substantial interactions between N and carbon content during crop production (Henriksen and Breland, 1999; Cheng et al., 2010; Ko et al., 2010; Zi et al., 2018). These findings allow us to reasonably conclude that N application, particularly NO_3_^–^-N and CO(NH_2_)_2,_ positively affected the total starch content in this study. Because none of the foliar N fertilizers significantly influenced the amylose content, we conclude that all three types of fertilizer promoted starch biosynthesis by increasing the amylopectin content. Our finding that grain sucrose levels were similar under both water conditions appears to contradict the observation that the SD treatment severely inhibited grain filling and starch accumulation. Based on pertinent sources (Ahmadi and Baker, 2001; Yang et al., 2014; Xu et al., 2016), we speculate that the sucrose content did not decrease under the SD treatment because severe drought stress hinders grain filling, decreases grain phytohormone levels, represses starch biosynthesis, and causes unconverted sucrose to accumulate.

To establish the effects of N fertilizer type on the expression levels of the genes encoding the enzymes involved in starch biosynthesis, we selected genes known to be regulated in wheat endosperm during grain filling (Yang et al., 2004b; Tetlow, 2006; Dai et al., 2008; Zhao et al., 2008; Cao et al., 2012; Kumar et al., 2018). The genes *AGPP-L, GBSSI, SSSI, SSSII, SSSIII, SBEI, SBEIIa*, and *SBEIIb* have various functions in starch biosynthesis, expressing different levels throughout grain filling. As substrate for the formation of starch, ADP-glucose is synthesized by the action of the AGPP enzyme encoded by *AGPP-L* in wheat (Jin et al., 2018). The gene *GBSSI* is reported to encode GBSS, which is involved in the formation of amylose; and *SSSI, SSSII, SSSIII, SBEI, SBEIIa*, and *SBEIIb* all play active roles in amylopectin synthesis by encoding SSS and SBE in wheat grain, respectively (Sarka and Dvoracek, 2017; Tetlow and Emes, 2017). Our results show that these genes were markedly upregulated by N fertilizer treatments at the specific period of grain filling, although the expression levels of *SSSIII* and *SBEI* did not significantly differ between the NH_4_^+^-N treatment and the control. The relative changes in expression level of these genes were consistent with the observed changes in amylose, amylopectin, and total starch content. We surmised that various types of N application may improve starch synthesis by regulating the expression of relevant genes, even under drought stress.

N fertilizer application influences endogenous phytohormone levels and regulates grain filling (Yang J. C. et al., 2001; Garnica et al., 2010; Kumar et al., 2018). In the present study, foliar applications of CO(NH_2_)_2_ and NO_3_^–^-N significantly increased Z+ZR and ABA and decreased ETH evolution during the early and middle grain filling stages. Zhang and Yang (2004) suggested that several hormones collectively regulate grain filling and starch biosynthesis. High grain ZR levels induce endosperm cell cleavage and are positively correlated with maximum grain weight and mean grain filling rate (Zhang et al., 2009). ABA regulates grain filling and starch biosynthesis by participating in the sugar-signaling pathway and enhancing the transport of stored assimilates, while ethylene inhibits grain filling in wheat and rice by promoting premature senescence (Yang et al., 2006; Zhu et al., 2011; Kumar et al., 2018). Lower ETH and higher ABA levels contribute to grain filling (Yang et al., 2007; Lv X. K. et al., 2017). Yang et al. (2006) suggest that the ratio of ABA/ETH is closely related to grian filling rate and sink capacity of wheat. We suggest that Z+ZR induced by foliar CO(NH_2_)_2_ and NO_3_^–^-N may have increased grain sink capacity by inducing endosperm cell division. The antagonism between ABA and ETH can be attenuated by CO(NH_2_)_2_ and NO_3_^–^-N application so that grain sink activity is increased. Furthermore, we observed that the genes upregulated by NO_3_^–^-N and CO(NH_2_)_2_ were positively correlated with ABA and ZR content and negatively correlated with ETH evolution rate (Table 2). Previous studies reported correlations between phytohormone levels and starch biosynthesis. ABA and other endogenous plant growth regulators control starch biosynthesis in wheat grains (Xie et al., 2003; Wang Z. et al., 2015; Xu et al., 2016; Kumar et al., 2018). Our results and previous reports indicate that under the foliar treatments of NO_3_^–^-N and CO(NH_2_)_2_, expression of genes encoding enzymes involved in starch synthesis can be regulated by endogenous phytohormone balances. This promotes wheat grain filling, whose mechanism may be the activation of carbohydrate transport, endosperm cell cleavage, and starch biosynthesis signaling (Xie et al., 2003; Albacete et al., 2014).

**TABLE 2:** Correlation coefficients between the expression of genes and the levels of hormones, the ratio of ABA/ETH in wheat grains.

**Genes**	**ZR**	**ABA**	**ETH**	**ABA/ETH**
*AGPP-L*	0.8468**	–0.1016	–0.1044	0.8335**
*AGPP-L*	0.8803**	–0.0425	–0.0663	0.9355**
*GBSSI*	0.5764**	0.4439*	−0.4237*	0.8758**
*SSSI*	0.9805**	–0.384	0.3096	0.7739**
*SSSII*	0.7644**	0.1575	–0.2043	0.9742**
*SSSIII*	−0.4456*	0.9305**	−0.8786**	0.1143
*SBEI*	0.9124**	0.0213	0.0057	0.8753**
*SBEIIa*	0.8462**	0.1238	–0.1103	0.9575**

Correlation is significant at the: *0.05 level, **0.01 level.

Our research demonstrated that duration of the grain filling period and grain weight are positively correlated to the activity of SOD, POD, CAT, and negatively correlated to the content of MDA (*R* = 0.9403^∗∗^, 0.9153^∗∗^, 0.9447^∗∗^, -0.8359^∗∗^, respectively). The duration of grain filling is closely related to plant senescence (Zhao et al., 2007). Plant senescence is a programmed process occurring late in the wheat growth period. It is hastened by abiotic stress, which induces the accumulation of reactive oxygen species (ROS) and MDA, and consequently shortens the duration of the grain filling period (Wang et al., 2013). ROS toxicity can be neutralized by antioxidant enzymes such as SOD, POD, and CAT, which protect nucleic acids, membrane proteins, and lipids in cells, and retard senescence in plants (Gregersen et al., 2008; Zhao et al., 2011). Our results indicate that foliar N application (especially NH_4_^+^-N and CO(NH_2_)_2_) can reduce scavenging ROS accumulation and protect cells by upregulating activity of SOD, POD, and CAT and lowering the content MDA in the flag leaves. When plants senesce, chlorophyll is lost, foliar photosynthesis declines, and structural chemicals degrade (Wang et al., 2019). At the same time, carbohydrate remobilization from the source (leaves) to the sink (grains) increases (Gregersen et al., 2013). Based on our observations, we conclude that drought stress induces premature senescence in plants by severely impairing biological activity through restricted photosynthate supply, accelerated carbohydrate remobilization to the grains, and a shortened grain filling period. We suggest that foliar N application (especially NH_4_^+^-N) at anthesis retards senescence by increasing photosynthate supply and delaying carbohydrate transformation. No significant differences were found among N applications (except NH_4_^+^-N) and the control in terms of grain filling period under drought stress, although the N treatments attenuated senescence. When wheat is subjected to water stress, the grain yield potential becomes limited so that remobilization of nutrients reserved in stems and leaves is critical to increasing grain yield (Yang and Zhang, 2006). We suggest that when severe drought stress restrains the photosynthate supply after anthesis, NO_3_^–^-N and CO(NH_2_)_2_ treatments promote remobilization of carbohydrates from source organs to grains for starch synthesis, which increases the grain filling rate and thereby accelerates plant senescence and shortens the grain filling period.

Overall, we found that drought stress severely suppresses wheat growth, restricts grain filling, and induces premature senescence (Wang X. et al., 2015; Djanaguiraman et al., 2018). Based on the foregoing analysis of grain phytohormones, starch biosynthesis, and leaf senescence, we propose that the three forms of foliar N fertilizer application mitigate drought stress-induced grain filling damage; however, the specific mechanisms involved differ for each N source. As drought stress seriously limits the source supply by impairing photosynthetic capacity, carbohydrates reserved before anthesis must be rapidly transported to the grain (Yang and Zhang, 2006). The gains from enhanced remobilization of the “reserve pool” and the accelerated grain filling rate may outweigh the reductions in photosynthesis and grain filling period duration, resulting in a net increase in grain yield (Schnyder, 1993; Yang and Zhang, 2006). This is supported by our observation that foliar application of NO_3_^–^-N produced the largest grain weight under drought stress. The differences in the regulatory effects of the three foliar N fertilizers may be explained by their different functional characteristics and metabolic pathways. It is known that nitrate and ammonium are absorbed into plant cells by transporters of the NRT family and AMT family, respectively (Liu et al., 2015). Nitrate and urea both must be converted to ammonia in order to be assimilated into amino acids (Andrews et al., 2013). According to Patterson et al. (2010), ∼40% of N-regulated genes are differentially expressed by either nitrate or ammonium in Arabidopsis thaliana plants. Nitrate itself can serve as a signal to regulate the response of cytokinins and the metabolism of N and carbon on the level of mRNA and proteins (Yoneyama and Suzuki, 2018; Mu and Luo, 2019). The ammonium-specific pattern of gene expression plays key roles in modulating extracellular acidification and downstream metabolites in the pathway of ammonium assimilation (Patterson et al., 2010). Omics technology may be a useful tool in further elucidating the regulation pathways of various N sources on grain filling, including hormones levels, starch synthesis and plant senescence. In addition, soil fertilizer levels also strongly influence wheat grain filling (Yan et al., 2019). Future field studies are needed to evaluate the effects of various foliar N fertilizer sources in conjunction with soil water in order to develop practical wheat crop management protocols.

## Conclusion

The present study showed that all three forms of foliar N application mitigated the deleterious effects of drought stress on wheat grain filling, although their modes of action differed. Foliar applications of CO(NH_2_)_2_ and NO_3_^–^-N controlled endogenous phytohormone activity in the grains, upregulated the genes involved in the sucrose-starch conversion pathway, and remobilized carbohydrates from the source organs (leaves). The sink strength and grain filling rate can be improved. In contrast, foliar NH_4_^+^-N application notably upregulated the antioxidant enzyme system, delayed senescence, hence the period of grain filling and photoassimilate supply was extended. Under water deficit, the relative differences in the three forms of nitrogen fertilizer in terms of their effect on grain filling were magnified. Improvement of carbohydrate transport and increase in sink strength were the key factors for grain weight increase. Therefore, foliar applications of CO(NH_2_)_2_ and NO_3_^–^-N were comparatively more efficacious at enhancing grain filling and the formation of grain weight under drought stress. Along with current research, molecular investigations are required for a better understanding of the functional characteristics and metabolic pathways of various nitrogen sources on grain filling, which will be helpful to optimize wheat grain yield and quality against climate change.

## Data Availability Statement

The raw data supporting the conclusions of this article will be made available by the authors, without undue reservation.

## Author Contributions

XW, YL, and XL conceived and designed the study. XL, YD, and ML performed the experiment. XL, WL, and XG collected and analyzed the data. XL wrote the manuscript. XW and YL revised the manuscript. All authors read and approved the final manuscript.

## Conflict of Interest

The authors declare that the research was conducted in the absence of any commercial or financial relationships that could be construed as a potential conflict of interest.

## Abbreviations

ABA: abscisic acid
AGPP: adenosine diphosphate pyrophosphorylase
CAT: catalase
ETH: ethylene
GBSS: granule-bound starch synthases
MDA: malondialdehyde
N: nitrogen
NH_4_^+^: ammonium
NO_3_^–^: nitrate
NUE: nitrogen use efficiency
POD: peroxidase
ROS: reactive oxygen species
SOD: superoxide dismutase
SBE: starch branching enzymes
SSS: soluble starch synthase
ZR: Z-riboside.
