# Supplementary material for: Effect of Foliar Application of Various Nitrogen Forms on Starch Accumulation and Grain Filling of Wheat (Triticum aestivum L.) Under Drought Stress
Source: Front Plant Sci. 2021 Mar 25;12:645379. doi: 10.3389/fpls.2021.645379 (PMC8030621; doi:10.3389/fpls.2021.645379)
Supplement: Supplementary file 1 [file Table_1.DOCX]

**Supplementary Table 1** The primer sequences of genes encoding starch synthase used for

| Name | NCBI No. | forward primer sequence | reserve primer sequence | bp |
| --- | --- | --- | --- | --- |
| *AGPP-L* | No. Z21969 | TTCGTTCACGCCTAAACTCCG | TGACCACGTCCCTTCCTATCC | 189 |
| *GBSSI* | No.AF286320 | TACGACCAGTACAAGGACGCC | GCAGTGGAAGTACCTCACCCTC | 90 |
| *SSSI* | No.AF091803 | TGGAACCCCACCACAGACA | AGGTAAACCCAGCTCCTTCTG | 105 |
| *SSSII* | No.AF155217 | CCGAGTTGCCTGAGCACTACCT | TCTTCCAGTCGTTCTGCCGTAT | 195 |
| *SSSIII* | No.AF258608 | TACTGTCGAGGTTATTCTCCCG | CGACTCGTCCAACCCATACTT | 122 |
| *SBEI* | No.AF286317 | TGGGCACAGGATGACAAAGG | TGGGCACAGGATGACAAAGG | 149 |
| *SBEIIa* | No. Y11282 | TGGCCTTAGACTCCGACGATG | TGCGGATGTTCGGTTGTGA | 85 |
| *SBEIIb* | No.AY740401 | ATTGACCCAACGCTCCGAGAC | ACCCATTCGCACCTTCACC | 339 |
| *β-actin* | No.AB181991 | AGCGGTCGAACAACTGGTA | AAACGAAGGATAGCATGAGGAAGC | 101 |

amplification
